# Counting the invisible: dietary inorganic phosphorus intake across different chronic kidney disease stages in elderly patients-a national insight

**DOI:** 10.1186/s41043-025-01141-5

**Published:** 2025-12-17

**Authors:** Amin Roshdy Soliman, Reham Abdelghany, Tarek Samy Abdelaziz, Abeer Attia, Rabab Mahmoud Ahmed

**Affiliations:** 1 Internal medicine and Nephrology- Kasr Alainy Faculty of medicine- Cairo University, Cairo, Egypt; 2 Department of public health and Community medicine- Kasr Alainy Faculty of medicine, Cairo University, Cairo, Egypt

**Keywords:** Chronic kidney disease, Elderly patients, Processed food, Inorganic phosphorus, Dietary patterns

## Abstract

**Background:**

There’s still a gap in research on phosphorus intake for elderly patients with chronic kidney disease. Processed and ultra-processed foods provide convenient dietary options but are significant source of harmful inorganic phosphorus. This study attempts to evaluate total phosphorus intake in elderly chronic kidney disease (CKD) patients across different stages, focusing on dietary contribution of inorganic phosphorus, stratified into tertiles, from processed and ultra-processed foods.

**Methods:**

Cross-sectional analytical study conducted using an adapted food frequency questionnaire to assess weekly consumption phosphorus patterns over the last year among 232 participants. The study investigates the frequency and types of natural high-phosphorus foods, as well as processed and ultra-processed foods commonly available in the Egyptian market. It focused on elderly chronic kidney disease patients aged over 65 years, covering various stages of chronic kidney disease, including those receiving haemodialysis, and compared their dietary intake to their caregivers and an age-matched group of individuals over 65 years without CKD.

**Results:**

Phosphorus intake among elderly CKD subgroups showed considerable inter-individuals difference, ranging 880 − 16,804 mg/week. The total median weekly phosphorus intake in CKD patients > 65 years was lower compared to caregivers, with a *statistically significant* difference (*p = 0.011*) for patients aged 65–74 years. Phosphorus intake declined as CKD progressed from stage II to V. Among CKD, patients with a diploma education& who cook for themselves were *significantly* more likely to exceed the Dietary Reference Intake, (*p < 0.05*).Weekly inorganic phosphorous intake showed significant proportionate with frailty score *(p = 0.024).* Phosphorus intake from processed food/UPF was categorized into tertiles (low (< 694.39 mg), moderate (694.39–2382.53 mg), and high (> 2382.53 mg). 41% CKD patients > 75 years were classified as low inorganic phosphorus consumers compared to 33.3% in CKD aged 65–74 years. Education level, BMI showed a *significant* association, *p < 0.05*. Patients undergoing three dialysis sessions per week consumed more than twice the inorganic phosphorus (1,314.9 mg) compared to those receiving two sessions (552.1 mg), *(p > 0.05)*. CKD patients > 75 years had *significantly lower* consumption of nuts, legumes, and beans than caregivers *(p = 0.048*).

**Conclusion:**

Age, CKD diagnosis and stage may be additional factors in the observed variations in phosphorus-containing food intake patterns.

**Supplementary Information:**

The online version contains supplementary material available at 10.1186/s41043-025-01141-5.

## Introduction

Hyperphosphatemia is a significant issue in CKD and dialysis patients in developing countries, affecting approximately 13% of population in Egypt [1]. Hyperphosphatemia from a potentially harmful source such as processed and ultra-processed foods (UPF), is associated with increased risk of CKD occurrence or progression and have been implicated in exacerbating pre-existing complications in individuals with CKD [2, 3].

Processed and UPF, while convenient and sometimes ensuring adequate caloric intake in the elderly, act as a double-edged weapon. On one hand, it provides easily accessible nutrients but on the other hand, the consumption of these types of foods presents significant risk for CKD patients attempting to restrict their phosphorus intake. This is because they contain high levels of inorganic phosphorus, which is absorbed almost entirely by the body [4]. Existing recommendations on dietary phosphorus intake sources do not specify a percentage for inorganic phosphorus and are largely based on expert opinions rather than strong epidemiological evidence [5]. Egypt’s hectic lives and urbanization are driving up demand for processed meals, beside other factors related to elderly such as socio-economic barriers, the physical limitation result in higher reliance on processed, phosphorus-rich foods despite awareness of the health risks associated with its intake [6]. Thus, evaluating the type and the source of phosphorus intake may be of importance.

CKD is more prevalent among adults aged 65 and older [7]. However, research on dietary phosphorus intake in elderly CKD patients across different stages-especially in Egypt-is lacking, particularly for non-dialysis patients. International studies also predominantly focus on haemodialysis patients, leaving a gap in understanding phosphorus intake across the broader CKD spectrum.

This study aimed to explore the estimated total/inorganic phosphorus consumption in Egyptian elderly CKD (at stage 2–5 including haemodialysis)/non-CKD patients matched age group and their younger caregivers and to categorizing phosphorus intake from processed foods into tertiles among the participants. Factors associated with their dietary phosphorus pattern will be assessed too.

*Our hypothesis*: The elderly CKD population had different dietary phosphorus pattern from caregiving and non-CKD elderly individuals due to their complex dietary needs.

## Methods

**Study design and participant recruitment **This cross-sectional analytical study, including elderly Egyptian CKD patients aged 65–74 years,>75 years with their care givers and age matched non chronic kidney disease (CKD) group > 65 years old. Participants were recruited from internal medicine out patients’ clinics at Cairo University Hospitals from January 2024 to April 2024, Egypt. The study protocol received approval from the Research Ethical Committee of Cairo University on November 11, 2023, with approval number N-434–2023. The study protocol conformed to ethical guidelines of the Declaration of Helsinki, 1975. Written informed consent was obtained from all participants prior to their inclusion in the study.

**Eligibility criteria** included elderly patients with chronic kidney disease (CKD) stages 2–5, regardless of aetiology or duration, including those on haemodialysis, with subgroups of patients aged > 65–74 years (*N* = 66) and > 75 years (*N* = 25). All haemodialysis patients included in this study receive hemodialysis 2–3 times weekly. A non-CKD comparative group of individuals aged > 65 years (*N* = 25) was included to investigate whether dietary patterns were specifically associated with a diagnosis of CKD. Caregivers under 50 years old, responsible for providing care or making medical decisions for elderly patients in the study (*N* = 116), were eligible to participate.

Participants were excluded from the study if they had: neurologic or cognitive disorder, an underlying phosphate-losing disease, on intensive dialysis regimens, patients on peritoneal dialysis, kidney transplant recipient, who are enteral tube fed and elderly or caregivers with limited spoken or written language skills.

### Data collection methods and tools

We conducted a survey with 232 participants. Patients and caregivers were individually interviewed face-to-face by one of the investigators to complete a printed survey with close ended questions to assess qualitative and quantitative weekly phosphorus intake. A semi quantitative food frequency questionnaire (FFQ), with 16 food items/groups adapted based on dietary sources of natural high-phosphorus-containing foods {fish, meat, poultry, (nuts, legumes, beans), grain consumption, and dairy products}, as well as processed and UPF commonly used and available in the Egyptian market (chips, Luncheon, Frozen meat, processed cheese, tuna, pudding, cake made from wheat or flour, Biscuits, and cola & soft drinks) [8] used to evaluate the individuals’ usual dietary intake. Standard food portion sizes were used to calculate daily food intake in grams or millilitre per day [9]. Participants were required to report their consumption frequency of an intended serving of each food item during the last year on a weekly basis. Fruits and vegetables categories were excluded as most of CKD and dialysis patients may avoid them due to its high-potassium content [10, 11].

Questionnaire validity was assessed as follows: content validity was ensured by reviewing relevant literature [12] and by including questions about the frequency of consumption of food items containing phosphorus, including processed foods. Face validity was established through consultations with four nephrologists, and the questionnaire was subsequently translated into Arabic. Additionally, the internal consistency of the frequency-based responses (times per week) for phosphorus-containing food items was assessed using the Intra-class Correlation Coefficient (ICC), which was also found to be 0.8, reflecting good consistency in participants’ responses.

A pilot testing of the questionnaire conducted on 10% of sample size calculated for exploring participants understanding and modifications done according to the participants’ response. Then the modified version used to gather data from the study participants. The questionnaire was distributed to all selected participants, and 100% response rate was achieved minimizes concerns related to non-response bias and enhances the reliability of the findings. The high engagement level may be attributed to effective communication, clear questionnaire design.

The survey collected information relating to participants demographic data including age, sex, education, weight, height, and CKD stage (if applicable), frailty scores and cognitive integrity were assessed by Clinical Frailty Scale (CFS) [13] and to the frequency, standardized portion sizes for food categories examined. The results for each food item were then multiplied by the amount of phosphorus in one serving, as determined by Egyptian food composition Table [14]. Total phosphorus consumption was estimated using predefined formulas in the electronic Excel version of the Egyptian food composition tables. The sum of these values provided the participant’s average weekly phosphorus consumption. The daily recommended phosphorus intake is 800 to 1,000 mg/day applies to all sources for chronic kidney disease/dialysis according to KDIGO guidelines [15].

In this study, phosphorus intake from processed food among studied patients will be categorized into tertiles to assess the factors associated with dietary patterns and phosphorus burden. Participants will be classified as *low consumers (< 694.39 mg)*,* middle consumers (694.39–2382.53 mg)*,* and high consumers (>2382.53 mg)* of phosphorus from processed food. This classification is derived based on previously published studies that used tertiles or quartiles to examine phosphorus intake and its health implications [16, 17].

The estimated glomerular filtration rate (eGFR) was calculated with the 2021 CKD-EPI (Chronic Kidney Disease Epidemiology Collaboration) formula: [eGFR = 142×min⁡(Scr/κ,1)^α^×max⁡(Scr/κ,1) ^−1.200^ × 0.9938 ^Age^× Sex Factor] [18].

*Variables*: Scr = serum creatinine (mg/dL), κ (kappa) = 0.7 for females, 0.9 for males, α (alpha) = − 0.241 for females, − 0.302 for males, Sex Factor = 1.012 for females, 1.000 for male, Age = age in years, min(Scr/κ, 1) = the smaller of Scr/κ or 1, max(Scr/κ, 1) = the larger of Scr/κ or 1.

CKD graded according to the KDOQI (Kidney Disease Outcomes Quality Initiative) [15].

**Main outcome measure: **Characterize the average weekly dietary phosphate intake from processed/UPF and from natural high phosphorus containing food in elderly patients with CKD and their caregivers/and non-CKD elderly by questionnaire.

**Sample size**: The sample size was calculated for our primary outcome. With a sample size of at least 77, we will have a power of 90% to assess the following hypothesis: Null hypothesis: dietary phosphate quantification in CKD patients will be within the recommended daily intake of ~ 1000 mg/day. Alternative hypothesis: dietary phosphate in CKD elderly patients is higher than the recommended intake; of ~ 1300 mg/day on average (SD: ~800) [16].

Used test: one sample means test. Significance level: 0.05. Equation used: N=(zα − zβ/δ)2. Where: Zα is the critical value of z for significance (for a two-tailed test, with α = 0.05, this would be the quantile Φ − 1 (0.975) = 1.956). zβ is the normal quantile for 1 − power. δ is the difference between the mean under the alternative hypothesis and the null value, divided by the standard deviation.

**Statistical methods**: Analysis of data was done by IBM computer using SPSS (statistical program for social science version 23) as follows: Description of quantitative variables such as Median and IQR minimum and maximum Shapiro test of normality used to check the normality of data. Description of qualitative variables as number and percentage. Chi-square test was used to compare qualitative variables between groups. Fisher exact test was used when one expected cell or more are less than 5. Pairwise comparison between groups with Bonferroni adjustment conducted for qualitative variables between groups. Kruskal Wallis test was used in parametric data (SD > 30%mean) with pairwise comparison between groups. Spearman correlation test to assess the association between quantitative variables. *P* ≤ 0.05 significant.

## Results

The study included a total of 232 participants, of them, 91 individuals diagnosed with chronic kidney disease (CKD) and 141 non-CKD individuals. The study population was further stratified based on age and CKD status into the following subgroups: 66 participants (28%) with CKD aged 65–74 years, 25 participants (10.8%) with CKD aged 75 years or older, 25 participants (10.8%) without CKD aged 65 years or older and 116 participants (50%) without CKD under the age of 50 years. Female gender was predominant in overall the sample (53.9%) with no sex difference between groups. Regarding education there were significantly higher percentage of secondary level in CKD patients above 75 years old than care givers (28% CKD above 75 versus 6.9% in care givers, **p value 0.029**)while intermediate education percentage were higher in all groups than care givers(15.2% in CKD above 65,28% CKD above 75,28% Non-CKD above 65 years &3.4% caregivers respectively; **p value = < 0.001**). University education percentage were higher in care givers than the other groups (39.4% in CKD above 65, 24% CKD above 75, 36% Non-CKD above 65 years &67.2% caregivers respectively; **p value = < 0.001**).

Frailty was predominant with higher scores in those with CKD aged 75 or older, **p= 0.024**. They had the lowest percentage of independent cooking (12.5% vs. 44.8% in caregivers). CKD patients over 75 and 65 relied more on cooking partners compared to caregivers (52.3%, 45.8% vs. 19.8%) and children (21.5%, 29.2% vs. 9.5%), **p < 0.05**.

BMI showed modest differences across groups, with non-CKD > 65 years having the highest median BMI, **p = 0.044**. Additional demographic and clinical characteristics are summarized in Table 1.

**Table 1:** Basic characteristics of studied group

	All *N* = 232	CKD > 65years *N* = 66	CKD > 75years *N* = 25	Non- CKD > 65 years *N* = 25	Normal Caregivers *N* = 116	*P* value
	*N* (%)	*N* (%)	*N* (%)	*N* (%)	*N* (%)	
**Age**						
Median (IQR)	56(35–68)	67(66–68)	77(76–80)	67(66–75)	*#$35(29–42)	**< 0.001**
Range	18–95	61–95	75–87	65–89	18–50	
**Sex**						
Male	107(46.1)	34(51.5)	16(64)	10(40)	47(40.5)	0.120
Female	125(53.9)	32(48.5)	9(36)	15(60)	69(59.5)	
	**Education level**					
Primary	7(3)	2(3)	1(4)	0(0)	4(3.4)	1
Secondary	25(10.8)	7(10.6)	7(28)b	3(12)	8(6.9)a	**0.029**
Intermediate	28(12.1)	10(15.2)	7(28)	7(28)	4(3.4)b	**< 0.001**
University	119(51.3)	26(39.4)	6(24)	9(36)	78(67.2)b	**< 0.001**
Diploma	53(22.8)	21(31.8)	4(16)	6(24)	22(19)	0.215
**BMI**						
Median(IQR)	27.3(23.8–30.5)	27(23.9–30.9)	28(23.8–29.8)	31(27–32.4.4)	$27(23.4–29.4)	**0.044**
Range	16.6–46.7	19.5–46.7	17.6–35.9	17.1–39.1	16.6–41.5	
**BMI categories**						
Under weight	4(1.7)	0(0)	1(4)	1(4.2)	2(1.7)	0.184
Normal	75(32.5)	22(33.3)	7(28)	2(8.3)b	44(37.9)a	**0.042**
Overweight	91(39.4)	26(39.4)	11(44)	9(37.5)	45(38.8)	0.965
Class I obesity	40(17.3)	13(19.7)	5(20)	8(33.3)	14(12.1)	0.074
Class II obesity	18(7.8)	4(6.1)	1(4)	4(16.7)	9(7.8)	0.367
Class III obesity	3(1.3)	1(1.5)	0(0)	0(0)	2(1.7)	1
**Frailty**						
Not Frail	88(75.9)	51(77.3)	16(64)	21(84)	…	0.235
Frail	28(24.1)	15(22.7)	9(36)	4(16)	…	
**Frailty score**						
Median(IQR)	2(2–2)	2(2–2)	*2(2–6)	2(2–2)		**0.024**
Range	1–8	1–8	2–8	1–8		
**CKD stage**						
CKD II	6(6.6)	6(9.1)	0(0)			0.183
CKD III	13(14.3)	13(19.7)	0(0)			**0.017**
CKD IV	8(8.8)	5(7.6)	3(12)			0.679
CKD V	3(3.3)	3(4.5)	0(0)			0.559
CKD v.HD	61(67)	39(59.1)	22(88)			**0.012**
**Frequency of dialysis**						
Two times/week	16(17.6)	9(23)	7(31.8)			0.409
Three times/week	45(49.5)	30(76.9)	15(68.2)			
**Prescribed phosphate binders**	52(57.1)	34(51.5)	18(72)	0(0)	0(0)	0.078
**The type of phosphate binder**						
sevelamer	53(69.7)	34(66.7)	19(76)	0(0)	0(0)	0.405
calcium carbonate	13(17.1)	10(19.6)	3(12)	0(0)	0(0)	0.526
calcium acetate	42(55.3)	30(58.8)	12(48)	0(0)	0(0)	0.373
**Who cooks at home?**						
You	81(35.2)	16(24.6)a	3(12.5)a	10(40)	52(44.8)b	**0.004**
partner	72(31.3)	32(49.2)a	10(41.7)a	7(28)	23(19.8)b	**< 0.001**
your child	37(16.1)	14(21.5)a	7(29.2)a	5(20)	11(9.5)b	**0.026**
your parents	26(11.2)	0(0.0)a	0(0.0)	0(0.0)	26(22.4)b	**< 0.001**
other relatives	9(3.9)	1(1.5)	3(12.5)	1(4)	4(3.4)	0.127

Test of significance for age, BMI and frailty score is Kruskal Wallis with pairwise comparison. *Statistically significant different from CKD > 65years # Statistically significant different from CKD > 75years. $Statistically significant different from Non-CKD > 65year. Test of significance for Qualitative variables is chi-square and fisher exact with Bonferroni adjustment for pairwise comparison. Subletters a is different from b. p value significant ≤ 0.05

## Estimated weekly phosphorus intake between the studied groups

Phosphorus intake among CKD subgroups showed considerable inter-individuals variability, ranging from 880 to 16,804 mg per week. However, the total median weekly phosphorus intake in CKD patients aged over 65 years was lower compared to caregivers, a statistically significant difference (**p = 0.011**) was observed only between patients aged 65–74 years (6,160 mg/week) and caregivers (8,902 mg/week), Table 2.

**Table 2:** Weekly phosphorus intake among studied group

	All	CKD > 65years	CKD > 75years	Non- CKD > 65 years	Normal Caregivers	*P* value
**Total weekly phosphorus intake**						
Median(IQR)	7673.25(4185.93–10528.3.93.3)	6160(3447–8986)	5939(3207–9269)	7673(3839–10469)	*8902(5172–11109)	**0.011**
Range	880–17130.25.25	1418–16,700	880–16804	2307–17,130	970–16683	
**Weekly phosphorus from processed food**						
Median(IQR)	1367.3(498–3021)	1168(537–2665.34.34)	926(332.12–2264.5.12.5)	828(339.8–2374.4.8.4)	1574(562.28–3675.6.28.6)	0.208
Range	30–8782.5.5	30–7775.1.1	60–6681.14.14	99.34–7004.88.34.88	37.5–8782.5.5.5	
**Percentage of phosphorus from processed food to total phosphorus intake**						
Median(IQR)	22.6(8.5–38.9)	27(12.4–37.9)	20(7.15–33.25)	9.7(6–32.3.3)	25.6(9.1–40.5)	0.34
Range	0.4–78.2	0.4–67.2	1.5–70.4	3.7–61.8	0.5–78.2	
**Source of processed food containing phosphorus**						
**plant source**	36(16)	7(11.1)	6(25)	6(24)	17(15)	0.246
**Animal source**	14(6.2)	5(7.9)	3(12.5)	3(12)	3(2.7)	0.06
**Mixed**	175(77.8)	51(81)	15(62.5)	16(64)	93(82.3)	0.053

Test of significance for total weekly phosphorus intake, weekly phosphorus from processed food and percentage of phosphorus from processed food to total phosphorus intake is Kruskal Wallis with pairwise comparison. *Statistically significant different from CKD > 65years # Statistically significant different from CKD > 75years. $Statistically significant different from Non-CKD > 65year. Test of significance for Qualitative variables is chi-square and fisher exact with Bonferroni adjustment for pairwise comparison. Subletters a is different from b. P value significant ≤ 0.05

There was no statistically significant difference in phosphorus intake from processed and UPF among the study groups. However, CKD patients had higher percentage of intake compared to non-CKD subgroups. Details on the proportion of phosphorus sources from processed foods and ultra-processed are provided in Table 2.

## Factors associated with phosphorus intake exceeding the dietary reference intake (DRI) among CKD patients

The majority of CKD patients who exceeded 7000 mg/week of phosphorus intake (from all sources) were classified as CKD stage V on haemodialysis; however, this association was not statistically significant (*p* > 0.05). There was no significant association with age, sex, BMI categories, CKD stage, frequency of dialysis, or frailty. However, patients with a diploma level of education were significantly more likely to exceed the DRI (18.9% among those consuming < 7000 mg vs. 37.8% among those consuming ≥ 7000 mg; **p = 0.045**). In addition, patients who cooked for themselves were significantly more likely to exceed the DRI (13.7% vs. 32.4%; **p = 0.035**). Conversely, when their children prepared food for them, phosphorus intake was more likely to remain within the DRI (18 35.3% vs. 8.1%; **p = 0.003)**, Table 3.

**Table 3:** Factors associated with exceeding 7000 mg/week (RDI) among CKD patients

		*Weekly Total phosphorous intake		*p* value
		Less than 7000 *N* = 53	> 7000 *N* = 37	
	Age(years)	**N(%)**	**N(%)**	
	Median(IQR)	68(66–76)	68(67–75)	0.741
	Range	61–95	65–84	
**Sex**	Male	28(52.8)	21(56.8)	0.713
	Female	25(47.2)	16(43.2)	
**Group**	CKD stage 2–5 (5D) patients aged > 65years	38(71.7)	28(75.7)	0.81
	CKD stage 2–5 (5D) patients aged > 75years	15(28.3)	9(24.3)	
**Education level"**	Primary	3(5.7)	0(0)	0.266
	Secondary	9(17)	5(13.5)	0.655
	Intermediate	11(20.8)	6(16.2)	0.588
	University	20(37.7)	12(32.4)	0.605
	Diploma	10(18.9)	14(37.8)	**0.045**
**BMI categories**	Under weight	1(1.9)	0(0)	1
	Normal	15(28.3)	14(37.8)	0.341
	Overweight	22(41.5)	14(37.8)	0.726
	Class I obesity	13(24.5)	5(13.5)	0.199
	Class II obesity	2(3.8)	3(8.1)	0.377
	Class III obesity	0(0)	1(2.7)	0.411
**CKD stage**				
	CKD II	4(7.5)	2(5.4)	1
	CKD III	7(13.2)	6(16.2)	0.69
	CKD IV	4(7.5)	3(8.1)	1
	CKD V	3(5.7)	0(0)	0.266
	CKD v.HD	35(66)	26(70.3)	0.672
**Frequency of dialysis**				
	2	10(28.6)	6(23.1)	0.629
	3	25(71.4)	20(76.9)	
**Frailty**				
	Not frail	37(69.8)	29(78.4)	0.366
	Frail	16(30.2)	8(21.6)	
**Who cooks at home?**				
	You	7(13.7)	12(32.4)	**0.035**
	partner	24(47.1)	20(54.1)	0.517
	your child	18(35.3)	3(8.1)	**0.003**

*one missed case

Test of significance for age is Mann Whitney U test. Test of significance for Qualitative variables is chi-square and fisher exact P value significant ≤ 0.05

### Estimated weekly phosphorus intake across different CKD stages

There are no statistically significant differences in estimated total or inorganic phosphorus intake among different CKD stages, including dialysis patients. However, there is a general trend of decreasing total phosphorus intake as CKD progresses from stage II to stage V, except for patients undergoing haemodialysis, who have a slightly higher median intake compared to CKD V patients.

CKD IV stage has the highest contribution of processed and ultra-processed foods to phosphorus intake (37.4%), followed by CKD V.HD (22.6%), *p* > 0.05.

Within patients undergoing dialysis, patients receiving three times per week dialysis sessions have slightly higher total phosphorus intake (6433.3 mg) compared to those on two sessions per week (6197.9 mg) but their phosphorus intake from processed and UPF (1314.9 mg) is more than double that of patients on two sessions per week (552.1 mg) (Table 4).

**Table 4:** Details comparison of weekly phosphorus intake across different CKD stages

CKD stage	weekly total phosphorus intake	weekly phosphorous from processed food	% of processed from total
**CKD II**			
Median(IQR)	7107.5(3175–11188.53.53)	1740.75(537–4898.02.02)	28.55(16.6–37.4)
Range	1813.5–15415.05.5.05	311.1–5760.5.1.5	5.4–41.3
**CKD III**			
Median(IQR)	7023.4(5172.8–8779.8.8.8)	1992.3(941.18–2615.14.18.14)	35.9(20.7–38.9)
Range	1949.7–9983.3.7.3	30–6136.5.5	0.4–64.8
**CKD IV**			
Median(IQR)	5867.75(2989.3–10418.35.3.35)	1593.45(650.25–3122.4.25.4)	37.4(17.15–38.75)
Range	1494.25–12708.25.25.25	91.84–4818.82.84.82	2.1–44.2
**CKD V**			
Median(IQR)	5807.5(3925.13–9672.4.13.4)	385.92(238–2833.58.58)	6.6(6.05–24.3)
Range	3086.25–12493.8.25.8	189–5182.32.32	6–41.5.5
**CKD v.HD**			
Median(IQR)	6363.7(3424.75–9817.3.75.3)	1168(499.1–2665.34.1.34)	22.6(11.4–34.9)
Range	880–16804.3.3	60–7775.1.1	1.5–70.4
**p value**	0.986	0.791	0.608
**Frequency of dialysis**			
**Two times/week**			
Median(IQR)	6197.9(3076.9–10126.9)	552.1(202.3–2000)	20(6.8–27)
Range	880–10918	60–5582.1.1	1.5–60
**Three times/week**			
Median(IQR)	6433.3(3424.8–9725.5.8.5)	1314.9(597.9–2818.5.9.5)	26(13.5–39.2)
Range	1418.3–16804.3.3.3	60–7775.1.1	1.6–70.4
**p value**	0.588	0.102	0.119

Test of significance for total weekly phosphorus intake, weekly phosphorus from processed food and percentage of phosphorus from processed food to total phosphorus intake is Kruskal Wallis

P value significant ≤ 0.05

Dialysis patients exhibited a trend toward higher consumption of certain processed and ultra-processed foods compared to non-dialysis individuals; however, the difference was not statistically significant (Table 5).

**Table 5:** Percentage distribution of dietary preferences for processed foods according to the Dialysis

	Dialysis		*p* value
	No	Yes	
	*N*(%)	*N*(%)	
**Chips**	14(46.7)	32(53.3)	0.551
**Luncheon**	7(23.3)	17(28.3)	0.613
**Processed cheese**	14(46.7)	31(50.8)	0.71
**Frozen meat**	11(36.7)	20(32.8)	0.714
**preserved fish or tuna**	11(36.7)	30(49.2)	0.259
**Cream caramel. pudding. or pudding**	9(31)	20(35.7)	0.666
**frequency/w Cake made from wheat**	6(21.4)	24(39.3)	0.097
**Cake made from flour**	15(50)	40(65.6)	0.153
**Biscuits made from flour**	10(33.3)	23(37.7)	0.683
**Pepsi or similar soft drinks**	14(46.7)	33(54.1)	0.505

Chi-square test p value significant ≤ 0.05

## Phosphorus intake distribution and factors associated with consumption among studied group: tertile classification

In this study, phosphorus intake from processed food was categorized into tertiles (low (< 694.39 mg), moderate (694.39–2382.53 mg), and high (> 2382.53 mg)). Among studied groups, non-CKD patients aged > 65 years showed the highest percentage in the low consumer group (48%), followed by CKD patients aged > 75 years (41.7%). In contrast, high consumers were predominantly normal caregivers (37.2%), followed by CKD patients aged > 65 years (33.3), with no significant differences across these groups (*p* > 0.05). Regarding sex, there was no significant variation between males and females across intake categories. Education level showed a significant association, particularly among those with secondary education, where the majority were in the low and moderate intake groups **(p = 0.017).** Frailty status showed a non-significant trend toward lower intake among frail individuals. BMI analysis revealed a significant association in Class I obesity, where lowest percentage were in high intake groups (13.5%; **p = 0.017**). Other BMI categories did not show significant differences. CKD stages, frequency of dialysis, and who cook were not significantly associated with phosphorus intake. However, participants who cooked for themselves tended to be high consumers, while those whose children prepared meals were more often in the low intake group (Table 6).

**Table 6:** Factors associated with level of dietary phosphorus consumption among studied group

	Low consumers(< 694.39)	Moderate consumers (694.39–2382.53.39.53)	High consumers (> 2382.53)	*p* value
**Group**	N(%)	N(%)	N(%)	
**CKD stage 2–5 (5D) patients aged > 65years**	21(33.3)	21(33.3)	21(33.3)	1
**CKD stage 2–5 (5D) patients aged > 75years**	10(41.7)	8(33.3)	6(25)	0.517
**Non-CKD patients aged > 65 years**	12(48)	7(28)	6(24)	0.248
**Normal Caregivers**	32(28.3)	39(34.5)	42(37.2)	0.245
**Age**				
**Median(IQR)**	65(36–69)	49(35–67)	48(35–67)	0.268
**Range**	20–95	21–89	18–80	
**Sex**				
**Male**	36(34.6)	34(32.7)	34(32.7)	0.931
**Female**	39(32.2)	41(33.9)	41(33.9)	
**Education level**				
**Primary**	2(28.6)	2(28.6)	3(42.9)	1
**Secondary**	11(44)a	12(48)a	2(8)b	**0.017**
**Intermediate**	11(40.7)	8(29.6)	8(29.6)	0.685
**University**	37(31.9)	40(34.5)	39(33.6)	0.883
**Diploma**	14(28)	13(26)	23(46)	0.096
**Frailty**				
**Not Frail**	28(33.3)	28(33.3)	28(33.3)	0.129
**Frail**	15(53.6)	8(28.6)	5(17.9)	
**BMI categories**				
**Under weight**	2(50)	0(0)	2(50)	0.55
**Normal**	27(37)	22(30.1)	24(32.9)	0.717
**Overweight**	26(28.6)	30(33)	35(38.5)	0.334
**Class I obesity**	15(40.5)a	17(45.9)a	5(13.5)b	**0.017**
**Class II obesity**	5(31.3)	4(25)	7(43.8)	0.636
**Class III obesity**	0(0)	1(33.3)	2(66.7)	0.55
**CKD stage**				
**CKD II**	2(33.3)	1(16.7)	3(50)	0.777
**CKD III**	3(23.1)	5(38.5)	5(38.5)	0.702
**CKD IV**	3(37.5)	2(25)	3(37.5)	1
**CKD V**	3(75)	0(0)	1(25)	0.319
**CKD v.HD**	20(33.9)	21(35.6)	18(30.5)	0.598
**Frequency of dialysis**				
**Twice**	8(53.3)	5(33.3)	2(13.3)	0.137
**3 times**	12(27.3)	16(36.4)	16(36.4)	
**Who cooks at home?**				
**You**	23(29.5)	24(30.8)	31(39.7)	0.361
**partner**	25(33.8)	24(32.4)	25(33.8)	0.984
**your child**	15(40.5)	12(32.4)	10(27)	0.52
**your parents**	6(24)	11(44)	8(32)	0.421
**Other relatives**	5(55.6)	3(33.3)	1(11.1)	0.203

Test of significance for age is Kruskal Wallis Test of significance for Qualitative variables is chi-square and fisher exact with Bonferroni adjustment for pairwise comparison. Subletters a is different from b. p value significant ≤ 0.05

### Analysis of natural high phosphorus-containing food consumption

Poultry, dairy products, fish and meat food items were the main contributors to dietary phosphorus intake. These food categories were consumed by more than 75% of the total samples and show comparable consumption rates between CKD and non-CKD groups.

While nuts, legumes, and beans are excellent plant-based protein sources, CKD patients over 75 years less consuming this foods compared to normal caregivers (44% vs. 71.6%, **p = 0.048**), (Supplementary File 1). Poultry is the only food category showing a significant difference between CKD and non-CKD individuals, with caregivers consuming it more frequently per week, p value = 0.001. Other food categories, including fish, meat, and plant-based sources of phosphorus, show similar intake (Supplementary File 2).

### Analysis of processed and UPF consumption

Most of these food categories show no statistically significant differences in consumption between CKD and non-CKD groups suggesting that these categories may be less commonly recognized as a source of phosphorus.

Cake made from flour and soft drinks being the most consumed items in CKD subgroups. However, compared to caregivers, CKD patients over 75 years and non-CKD individuals over 65 years exhibited the lowest consumption of chips (32% and 37.5%, respectively), whereas normal caregivers had the highest intake (68.5%, **p = 0.001**). Similarly, Pepsi or similar soft drinks showed the lowest consumption in CKD patients over 75 years (44%) and the highest in non-CKD individuals over 65 years (75%, **p = 0.004**) (Supplementary File 1).

CKD patients tend to consume processed cheese less frequently/week, likely due to its high phosphorus content (**p = 0.027**), whereas non-CKD individuals over 65 years show the highest biscuit consumption frequency (**p = 0.002**) (Supplementary File 2).

Weekly mean total phosphorous intake showed significant positive correlation with frequency of eating natural high phosphorus containing food as well with processed and ultra-processed food per week. However, weekly phosphorous intake from processed and ultra-processed food showed significant proportionate with frailty score and frequency of processed food and ultra-processed per week (Table 7).

**Table 7:** The association between weekly phosphorus intake and weekly phosphorous intake from proceed food with other quantitative variable

All	Weekly phosphorus intake		Weekly phosphorus from processed food	
	**r**	**p value**	**r**	**p value**
Weekly phosphorus from processed food	0.631**	**< 0.001**		
BMI	−0.045	0.497	−0.002	0.975
Frailty score	0.008	0.935	− 0.198*	**0.036**
Times of eating fish in a week	0.228**	**0.002**	.	
Times of eating poultry in a week	0.428**	**< 0.001**		
Times of eating meat in a week	0.117	0.103		
Times of eating nuts. legumes or beans in a week	0.260**	**0.001**		
Times of eating Grains (Bran flakes or instant Oatmeal) in a week	0.315**	**< 0.001**		
Times of eating a week you eat dairy product in a week	−0.078	0.26		
Times of eating Chips in a week	0.423**	**< 0.001**	0.379**	**< 0.001**
Times of eating luncheon in a week	0.355**	**0.001**	0.436**	**< 0.001**
Times of eating Processed cheese in a week	0.243*	**0.012**	0.323**	**0.001**
Times of eating Frozen meat in a week	0.231*	**0.039**	0.392**	**< 0.001**
Times of eating preserved fish or tuna in a week	0.324**	**0.001**	0.517**	**< 0.001**
Times of eating Cream caramel. pudding. or pudding in a week	0.362**	**0.002**	0.299*	**0.01**
Times of eating Cake made from wheat in a week	0.05	0.677	0.246*	**0.036**
Times of eating Cake made from flour in a week	0.173*	**0.039**	0.162	0.052
Times of eating Biscuits made from flour in a week	0.058	0.568	0.158	0.115
Times of eating Pepsi or similar soft drinks in a week	0.239**	**0.003**	0.387**	**< 0.001**
Frequency of binders intake/day	−0.147	0.209	−0.089	0.449

Spearman correlation test P value significant ≤ 0.05

## Discussion

This study addresses a critical knowledge gap in CKD management by providing the first comprehensive analysis of phosphorus intake patterns across all CKD stages in elderly Egyptian patients. Given that phosphorus management is fundamental to preventing cardiovascular complications and bone disease progression in CKD; these findings have immediate clinical relevance for the Egypt’s population affected by CKD [2].

Managing phosphorus intake in CKD patients is complicated due to several factors. The complex diet makes tracking phosphorus difficult, while eating out introduces hidden sources. Portion estimation errors lead to overconsumption, and CKD-friendly foods estimated 2–3 times cost of regular alternatives based on local market survey, creating socio-economic barrier to adherence. Limited availability of convenient, kidney-friendly options in grocery stores further complicates adherence to dietary guidelines, pushing patients toward suboptimal choices [19].

Our findings reveal that while the mean weekly phosphorus intake from all sources aligned with recommendations (5,600–7,000 mg/week) in half of elderly CKD patients, inter-individuals difference (880–16,804 mg/week) represents the difference between optimal management and levels associated with increased mortality risk in CKD patients and indicates that a subset consumed phosphorus far exceeding guidelines. Phosphorus intake declined progressively with CKD stage advancement, indicating adaptive dietary adjustments to disease severity. Protein intake becomes more restricted, while chronic inflammation and hormonal imbalances increasingly affect appetite and nutrient absorption as CKD advances. However, dialysis patients exhibited a paradoxical increase in intake, potentially due to perceived reduced dietary restrictions post-dialysis initiation. This trend was particularly obvious in our study among individuals undergoing dialysis three times per week compared to those receiving two sessions per week [20].

*Dietary patterns across CKD stages*: No significant variation in processed and UPF derived phosphorus across CKD stages or between studied groups were found in our study. This finding challenge the generalizability of the hypothesis that older individuals typically consume more processed foods and contrasts with the notion that most elderly individuals’ eating habits remain largely consistent with those established earlier in life [21], indicate that additional factors may influence processed and ultra-processed food intake and reflect the profound impact of disease diagnosis on health vigilance.

*Cultural and socio-economic contexts*: In our study, the 9–27% contribution of processed foods to total phosphorus intake in our Egyptian cohort contrasts sharply with Western populations (44% in US, 51% in Europe), suggesting distinct dietary preservation of traditional eating patterns and cultural preference of fresh ingredients despite CKD diagnosis. This finding challenges assumptions about global dietary convergence and highlights the importance of region-specific nutritional guideline [22–24].

*Implications for clinical practice*: In this research, education level, frailty score and BMI were associated with high phosphorus consumption and this matches the results of previous studies [25–28]. CKD patients aged 65–74 consumed more phosphorus than those over 75, aligning with Alexandria’s and north Africa haemodialysis cohort with hyperphosphatemia [2, 29]. Younger patients also had a higher intake of processed and UPF, possibly due to a preference for fast foods containing inorganic phosphorus additives [30]. Dialysis patients frequently consumed processed foods, such as baked goods and soft drinks, during intra-dialytic snacking, a pattern consistent with findings from previous studies [31–33]. This contrasts with CKD stage V patients on conservative therapy, who exhibited the lowest processed food intake likely due to stricter medical supervision [34].

Caregivers and non-CKD elderly usually consume larger portion sizes and a broader range of foods. Their dietary pattern observed in our study was matched with findings from recent researches on general population in Egypt [6, 35–37]. This pattern deserves further investigation as it may represent a risk factor for future development of metabolic disorders including CKD.

Egypt’s traditional diet heavily relies on legumes, whole grains, and fresh vegetables with less reliance on processed foods. Interest in plant-based eating and alternative proteins with affordable prices is the usual scenario in Egypt and Middle East region [38].

As regards nutritional pattern in Elderly CKD patients Vs. dietary needs in those patients, we found that most of them relies on animal based with high protein to phosphorus ratio sources compared to less consumption of legumes, beans and nuts despite its availability in Egypt [39], which does not seem to directly match traditional Egyptian dietary habits and to the low in sodium, phosphorus, and protein intake recommended for CKD patients. This may be due to legumes, beans and nuts also contain potassium, which CKD patients must often limit to prevent electrolyte imbalances or those patients may experience difficulty chewing or fibres digestion.

This pattern aligns partly with modern urban diets, it blend traditional and modern diets, with varying levels of processed food consumption where animal-based proteins are prioritized, processed foods are present, and plant-based nutrition is secondary among certain populations. It shares similarities with Asian and Iranian diets, but differs from Mediterranean, Brazilian, and DASH-style diets [39].

Part of our patients (HD patients) may benefit from this pattern as limiting dietary protein intake to manage serum phosphorus may have more risks than benefits and increase mortality rates for maintenance haemodialysis (MHD) patients as indicated in a recent research [40]. CKD patients in Egypt may face malnutrition due to dietary restrictions and inflammation, requiring careful nutritional planning. On other hand, Studies indicate that adherence to Mediterranean, DASH or healthier diets may benefit kidney health, while Western-style diets may increase CKD progression risk.

The differences in consumption patterns between CKD and Non-CKD elderly groups warrant longitudinal investigation to determine whether these dietary habits precede or follow CKD diagnosis.

*The strengths* of this study: it provides classification for of phosphorus intake from processed and UPF in elderly CKD patients. Intake pattern was studied in All CKD stages including haemodialysis patients. The utilization of multiple food categorizations facilitated the analysis of general dietary trends among elderly patients with chronic kidney disease (CKD), as well as insights into phosphorus consumption patterns in elderly individuals without CKD and younger populations. This approach broadens the applicability of the findings to a large segment of the Egyptian population and provides up-to-date dietary data.

*Limitations*: this study has several limitations including: reliance on self-reported dietary intake which may be subject to recall bias; the cross-sectional design which cannot establish causality whether observed dietary patterns are adaptive responses to CKD progression or pre-existing habits that contributed to disease development. Longitudinal follow-up is essential to establish temporal relationships and inform preventive strategies; limited generalizability beyond the Egyptian context, seasonal dietary variations common in Mediterranean climates were not captured in this single-time point assessment, Portion size estimation errors, common in elderly populations, may compound intake miscalculations and potential underestimation of actual phosphorus intake due to incomplete food composition databases for local processed and ultra-processed foods. The study does not explore how urbanization may confound findings which warrant deeper analysis. Serum phosphorus levels and phosphorus binder usage were not correlated with dietary intake, limiting assessment of the clinical effectiveness of current dietary management strategies. Despite limitations, this study provides essential baseline data for developing evidence-based, culturally-appropriate phosphorus management strategies for elderly Egyptian CKD patients, with immediate implications for clinical practice and longer-term population health planning.

*Future Directions*: longitudinal design to track dietary adaptation over CKD progression, rural population inclusion, seasonal dietary variation assessment, and cost-effectiveness analysis of culturally-adapted dietary interventions.

**In conclusion**

Main contributor to the dietary phosphorus intake in elderly CKD patients is animal based, fresh source (poultry). The second largest dietary phosphorus sources after poultry were dairy products. This survey reported a reduction in plant based natural phosphorus containing food. A considerable number of elder CKD patients continue to experience problems restricting dietary phosphorus indicating inconsistent counselling approach. 9–27% of consumed phosphorus comes in elderly CKD Egyptian patients from inorganic source. Higher processed food consumption among dialysis patients indicating a need for focused nutritional education on dialysis initiation. Patients who cook independently exceed phosphorus recommendations more frequently than those whose children prepare meals suggesting that, family involvement in nutritional care may be protective. This has particular relevance in cultures like Egypt where people will overlook their own desires with meals collectively shared, rather than individuals undertaking. With continued urbanization, the percentage of processed food may increase potentially worsening phosphorus management in future CKD population.

## Supplementary Information

Supplementary Material 1

Supplementary Material 2

## Data Availability

No datasets were generated or analysed during the current study.

## Abbreviations

CFS: Clinical frailty scale
CKD: Chronic kidney disease
DASH: Dietary approaches to stop hypertension
DRI: Dietary reference intake
eGFR: Estimated glomerular filtration rate
HD: Hemodialysis
ICC: Intra-class correlation coefficient
KDIGO: Kidney disease improving global outcomes
KDOQI: Kidney disease outcomes quality initiative
MHD: Maintenance haemodialysis
NHANES: National health and nutrition examination survey
UPF: Ultra-processed foods
