# Supplementary material for: Counting the invisible: dietary inorganic phosphorus intake across different chronic kidney disease stages in elderly patients-a national insight
Source: J Health Popul Nutr. 2025 Dec 17;45:24. doi: 10.1186/s41043-025-01141-5 (PMC12821956; doi:10.1186/s41043-025-01141-5)
Supplement: Supplementary file 1 — Supplementary Material 1 [file 41043_2025_1141_MOESM1_ESM.docx]

**Supplementary file 1: Comparison of nutrition pattern from natural high phosphorus containing food and processed food/UPF between the studied groups**

|  | **All** | | **CKD >65years** | **CKD >75years** | **Non- CKD > 65 years** | **Normal Caregivers** | **p value** |
| --- | --- | --- | --- | --- | --- | --- | --- |
| **Nutrition pattern from high phosphorus containing food** | **N (%)** | **N (%)** | | **N (%)** | **N (%)** | **N (%)** |  |
| Fish | 195(84.4) | | 57(86.4) | 20(80) | 19(76) | 99(86.1) | 0.507 |
| Poultry | 221(95.3) | | 63(95.5) | 22(88) | 25(100) | 111(95.7) | 0.275 |
| Meat | 183(78.9) | | 56(84.8) | 18(72) | 19(76) | 90(77.6) | 0.501 |
| Nuts. Legumes or beans | 149(64.2) | | 39(59.1) | 11(44)b | 16(64) | 83(71.6)a | **0.048** |
| Grains (Bran flakes or instant Oatmeal) | 173(74.6) | | 45(68.2) | 18(72) | 21(84) | 89(76.7) | 0.397 |
| Dairy product | 209(90.5) | | 60(90.9) | 24(96) | 22(88) | 103(89.6) | 0.8 |
| **Nutrition pattern from processed food containing**  **High phosphorus** | | | | | | | |
| Chips | 129(58.1) | | 38(58.5) | 8(32)b | 9(37.5)b | 74(68.5)a | **0.001** |
| Luncheon | 77(34.1) | | 17(26.2) | 7(28) | 7(29.2) | 46(41.1) | 0.177 |
| Processed cheese | 106(45.7) | | 32(48.5) | 13(52) | 9(36) | 52(44.8) | 0.662 |
| Preserved fish or tuna | 112(48.3) | | 30(45.5) | 11(44) | 10(40) | 0(0) | 0.584 |
| Cream caramel. Pudding. Or pudding | 73(32.3) | | 7(29.2) | 8(32) | 36(31) | 61(52.6) | 0.9 |
| Cake made from wheat | 73(31.9) | | 12(48) | 9(36) | 34(29.6) | 36(31) | 0.274 |
| Cake made from flour | 144(62.1) | | 17(68) | 14(56) | 75(64.7) | 34(29.6) | 0.646 |
| Biscuits made from flour | 101(43.5) | | 9(36) | 13(52) | 55(47.4) | 75(64.7) | 0.334 |
| Pepsi or similar soft drinks | 149(64.2) | | 11(44)a | 15(60) | 87(75)b | 55(47.4) | **0.004** |
| Frozen meat | 80(34.5) | | 25(38) | 6(24) | 5(20) | 44(37.9) | 0.214 |

Test of significance for Qualitative variables is chi-square and fisher exact with Bonferroni adjustment for pairwise comparison. Subletters (a) is statistically significant different from (b) p value significant ≤0.05
