# Supplementary material for: Counting the invisible: dietary inorganic phosphorus intake across different chronic kidney disease stages in elderly patients-a national insight
Source: J Health Popul Nutr. 2025 Dec 17;45:24. doi: 10.1186/s41043-025-01141-5 (PMC12821956; doi:10.1186/s41043-025-01141-5)
Supplement: Supplementary file 2 — Supplementary Material 2 [file 41043_2025_1141_MOESM2_ESM.docx]

|  | **All** | **CKD >65years** | **CKD >75years** | **Non- CKD > 65 years** | **Normal Caregivers** | **P value** |
| --- | --- | --- | --- | --- | --- | --- |
| **Times of eating fish in a week** | | |  |  |  |  |
| Median(IQR) | 1(1-1) | 1(1-1) | 1(1-1) | 1(1-1) | 1(1-2) | 0.251 |
| Range | 0.25-3 | 0-3 | 1-3 | 0-2 | 0-3 |  |
| **Times of eating poultry in a week** | | |  |  |  |  |
| Median(IQR) | 2(2-3) | 2(1-3) | 3(1-3) | 2(2-3) | *3(2-4) | **0.001** |
| Range | 0.25-7 | 0.25-5 | 1-7 | 1-4 | 1-7 |  |
| **Times of eating meat in a week** | | |  |  |  |  |
| Median(IQR) | 1(1-2) | 1(1-2) | 1(1-1) | 2(1-2) | 1(1-2) | 0.087 |
| Range | 0.25-7 | 0.25-5 | 0.25-4 | 1-4 | 0.25-7 |  |
| **Times of eating nuts. legumes or beans in a week** | | | | |  |  |
| Median(IQR) | 1(1-2) | 1(1-2) | 1(1-2) | 1(1-2) | 1(1-2) | 0.928 |
| Range | 0.09-7 | 0.25-4 | 0.25-7 | 0.085-4 | 0.085-7 |  |
| **Times of eating Grains (Bran flakes or instant Oatmeal) in a week** | | | | | |  |
| Median(IQR) | 2(1-4) | 2(1-4) | 2(1-3) | 3(1-4) | 2(1-3) | 0.735 |
| Range | 0.25-7 | 0.5-7 | 1-7 | 1-7 | 0.25-7 |  |
| **Times of eating dairy product** | | | |  |  |  |
| Median(IQR) | 1(1-1) | 1(1-1) | 1(1-1) | 1(1-1) | 1(1-1) | 0.134 |
| Range | 1-7 | 1-7 | 1-2 | 1-7 | 1-7 |  |

**Supplementary file 2: Frequency of food items among studied groups per week**

|  | **All** | **CKD >65years** | **CKD >75years** | **Non- CKD > 65 years** | **Normal Caregivers** | **P value** |
| --- | --- | --- | --- | --- | --- | --- |
| **Times of eating Chips:** | | | | | | |
| Median(IQR) | 2(1-3) | 1(1-3) | 1(1-1) | 2(1-4) | 2(1-3) | 0.058 |
| Range | 1-7 | 1-7 | 1-2 | 1-7 | 1-7 |  |
| **Times of eating Luncheon** | |  |  |  |  |  |
| Median(IQR) | 1(1-2) | 1(1-2) | 1(1-2) | 1(1-2.5) | 2(1-2) | 0.549 |
| Range | 0.25-7 | 1-7 | 0.25-2 | 1-3 | 0.25-6 |  |
| **Times of eating Processed cheese** | |  |  |  |  |  |
| Median(IQR) | 2(1-3) | 1(1-2) | 1(1-2) | 2(2-3) | 2(1-3) | **0.027** |
| Range | 0.25-7 | 0.25-7 | 1-3 | 1-7 | 1-7 |  |
| **Times of eating frozen meat** | |  |  |  |  |  |
| Median(IQR) | 1(1-2) | 1(1-2) | 1(1-2) | 1(1-1) | 1(1-2) | 0.505 |
| Range | 0.25-7 | 1-7 | 0.25-2 | 1-3 | 1-7 |  |
| **Times of eating preserved fish or tuna:** | |  |  |  |  |  |
| Median(IQR) | 1(1-2) | 1(1-1) | 1(0.25-2) | 1(1-2) | 1(1-2) | 0.695 |
| Range | 0.25-5 | 0.25-5 | 0.25-3 | 1-3 | 0.25-4 |  |
| **Times of eating Cream caramel. pudding. or pudding** | | | |  |  |  |
| Median(IQR) | 1(1-2) | 1(1-2) | 1(0.25-2) | 2(1-3) | 1(1-2) | 0.189 |
| Range | 0.25-7 | 0.25-3 | 0.25-4 | 1-7 | 1-4 |  |
| **Times of eating Cake made from wheat** | |  |  |  |  |  |
| Median(IQR) | 1(1-2) | 1(1-2) | 1(1-1) | 1(1-2) | 1(1-2) | 0.244 |
| Range | 0.25-7 | 0.25-7 | 0.25-3 | 0.25-7 | 1-7 |  |
| **Times of eating Cake made from flour** | |  |  |  |  |  |
| Median(IQR) | 1(1-2) | 1(1-2) | 1(1-2) | 1(1-2) | 1(1-2) | 0.691 |
| Range | 0.25-7 | 0.5-6 | 1-4 | 0.25-7 | 0.25-7 |  |
| **Times of eating Biscuits made from flour** | |  |  |  |  |  |
| Median(IQR) | 1(1-2) | 1(1-3) | 1(1-3) | *3(2-4) | *1(1-2) | **0.002** |
| Range | 1-7 | 1-7 | 1-4 | 1-6 | 1-7 |  |
| **Times of eating Pepsi or similar soft drinks** | | |  |  |  |  |
| Median(IQR) | 2(1-3) | 2(1-3) | 1(1-2) | 3(1-4) | 2(1-3) | 0.293 |
| Range | 0.5-7 | 1-7 | 0.5-7 | 1-7 | 0.5-7 |  |
